# Postoperative detection of pulmonary artery catheter entrapment during minimally invasive mitral valve repair: a case report

**DOI:** 10.1186/s40981-025-00822-8

**Published:** 2025-10-16

**Authors:** Ayaka Higashi, Sachiko Yamazaki, Atsushi Kainuma, Toshihito Mihara, Yuya Takahashi, Hiroki Matsuyama, Akiyuki Takahashi, Masahiro Sakaguchi

**Affiliations:** 1 Department of Anesthesiology, Kyoto Prefectural University of Medicine, Kyoto, Japan; 2 Department of Cardiovascular Surgery, Japanese Red Cross Kyoto Daiichi Hospital, Kyoto, Japan; 3 Department of Anesthesiology, Japanese Red Cross Kyoto Daiichi Hospital, Kyoto, Japan

**Keywords:** Pulmonary artery catheter, Pulmonary artery catheter entrapment, Minimally invasive cardiac surgery

## Abstract

**Background:**

Pulmonary artery catheter (PAC) entrapment is a rare but serious complication caused by inadvertent suturing to cardiac or vascular structures.

**Case presentation:**

A 42-year-old man underwent minimally invasive mitral valve repair via right mini-thoracotomy. Thirty minutes after weaning from cardiopulmonary bypass (CPB), pulmonary artery pressure suddenly dropped, and the waveform became identical to that of central venous pressure. Two centimeters of PAC mobility, aspiration of blood from the balloon port, and blood accumulation in the monitor connector were noted. On postoperative day 1, resistance was encountered during catheter withdrawal at 7 cm, and chest radiography revealed abnormal catheter curvature. Reoperation under CPB confirmed PAC entrapment at the left atriotomy suture line, and the catheter was successfully removed.

**Conclusions:**

In minimally invasive mitral valve repair, a left atriotomy approach might be associated with a risk of PAC entrapment, and the option of not inserting a PAC should always be considered.

## Introduction

A pulmonary artery catheter (PAC) is widely utilized during cardiac surgery due to its ability to provide continuous hemodynamic monitoring. Although PAC is a valuable tool, it carries risks such as arrhythmias, damage to valve leaflets, pulmonary artery rupture, infection from the indwelling catheters, and, in rare cases, catheter entrapment [1–13]. Entrapment of the PAC is a rare but serious complication in which the catheter becomes unintentionally sutured to cardiac or vascular structures. Recent studies show that its incidence is approximately 0.07%; however, the true incidence may be underestimated due to reporting and publication bias [1, 2].

Minimally invasive cardiac surgery (MICS) is becoming increasingly popular, as it may reduce short-term complications and improve cosmetic outcomes and recovery times [14]. However, MICS presents unique technical and anesthetic challenges that require specialized knowledge and skills [15]. For example, the limited surgical field and restricted instrument maneuverability in MICS may increase the risk of inadvertently suturing a catheter. In this case, we encountered PAC entrapment during a right mini-thoracotomy mitral valvuloplasty for mitral regurgitation (MR).

## Case presentation

A 42-year-old man (179 cm, 53 kg) with no significant medical history was diagnosed with MR due to A2–A3 prolapse and was scheduled to undergo minimally invasive mitral valve repair via a right mini-thoracotomy. Preoperative evaluation showed a left ventricular ejection fraction of 76%, no regional wall motion abnormalities, and no significant coronary artery stenosis on coronary angiography.

General anesthesia was induced uneventfully, and the patient was intubated with a 37 Fr double-lumen endotracheal tube. A central venous catheter (CVC), an introducer sheath, and a 7.5 Fr PAC were inserted via the right internal jugular vein. The CVC was placed at 13 cm, and the PAC was advanced to 50 cm under fluoroscopic and pressure waveform guidance without difficulty. The catheter tip was confirmed to be in the right pulmonary artery using transesophageal echocardiography (TEE).

The patient was positioned in the left lateral decubitus position. A 7 cm skin incision was made just lateral to the right nipple, extending dorsally. A right thoracotomy was performed through the third intercostal space under one-lung ventilation. Cardiopulmonary bypass (CPB) was established via femoral artery cannulation with venous drainage from the right femoral vein and the superior vena cava (SVC). The ascending aorta was cross‑clamped, and antegrade cardioplegia was delivered to achieve cardiac arrest. The mitral valve was exposed and repaired through a right-sided left atriotomy. Post-repair TEE showed only trivial MR and no evidence of stenosis.

Thirty minutes after weaning from CPB, pulmonary artery pressure (PAP) suddenly dropped; the mean PAP decreased from 20 to 10 mmHg. The pressure and waveform became identical to those of central venous pressure (CVP). At that time, under continuous infusions of norepinephrine (0.02 µg/kg/min), dobutamine (0.9 µg/kg/min), and nitroglycerin (0.3 µg/kg/min), the vital signs changed from heart rate (HR) 126/min, arterial blood pressure (ABP) 95/50 mmHg, CVP 10 mmHg, and PAP 22/18 mmHg to HR 109/min, ABP 91/52 mmHg, CVP 10 mmHg, and PAP 12/8 mmHg. Balloon inflation was attempted to verify the PAC tip position; however, the balloon failed to inflate, and the tip position could not be confirmed by TEE. The catheter was withdrawn by 2 cm without resistance or waveform change, and blood reflux was observed in the inflation syringe. After discussion with the surgeons, balloon rupture was suspected. The PAC was left in place, and the procedure was continued. The operative time was 7 h and 33 min. No allogeneic blood transfusion was required during the operation. After completion of the surgery, a small amount of blood was noted in the thermistor connector upon disconnection of the temperature cable. The patient was transferred to the intensive care unit (ICU) under sedation and intubation, and was extubated on postoperative day 1, as respiratory and hemodynamic parameters remained stable.

**Fig. 1:**
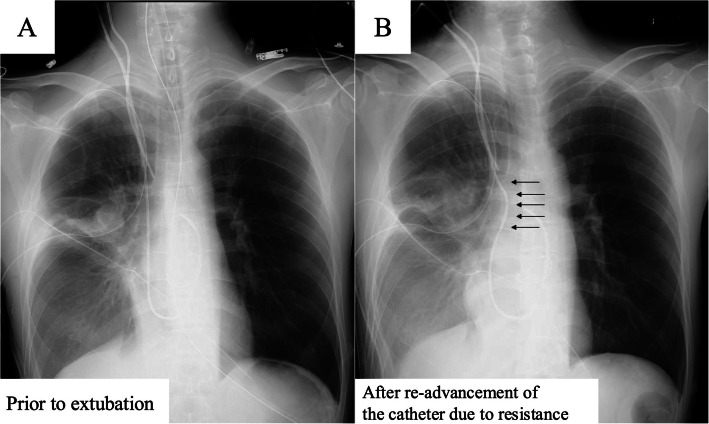
Pulmonary artery catheter positioning on chest radiographs. **A** Prior to extubation. **B** After re-advancement of the Pulmonary artery catheter due to resistance. Black arrow indicates abnormal curvature of the Pulmonary artery catheter near the superior vena cava

After extubation, removal of the PAC was attempted; however, resistance was encountered after approximately 7 cm of withdrawal. The PAC was returned to the initial insertion depth, and chest radiography (CXR) was performed. On CXR, compared with the CXR obtained prior to extubation, the PAC appeared abnormally curved near the SVC (Fig. 1). Based on these findings, suture entrapment of the PAC was suspected, and an emergent reoperation under general anesthesia was scheduled for catheter removal.

**Fig. 2:**
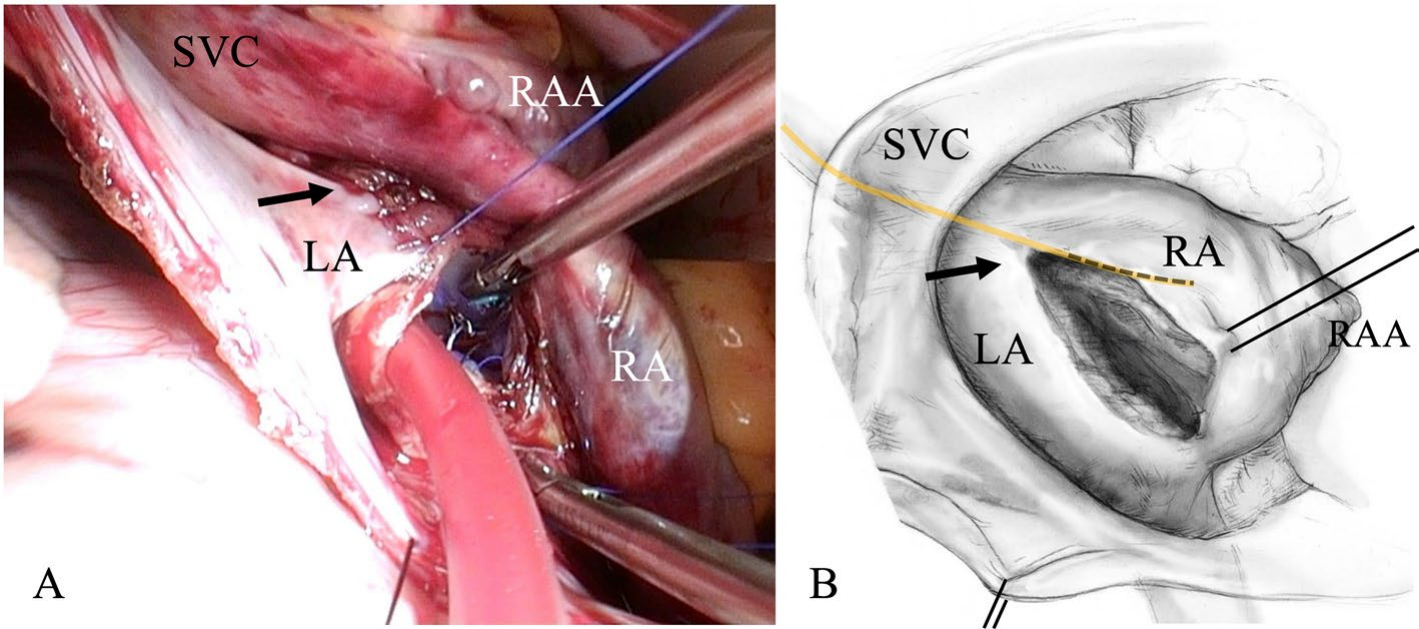
**A** Intraoperative image during left atrial closure in the first surgery. The black arrow indicates the suspected site of pulmonary artery catheter (PAC) entrapment. **B** Schematic illustration of panel **A**. The yellow and black dotted line indicates the suspected course of the PAC. The black arrow indicates the suspected site of PAC entrapment. Abbreviations: LA, left atrium; RA, right atrium; RAA, right atrial appendage; SVC, superior vena cava

**Fig. 3:**
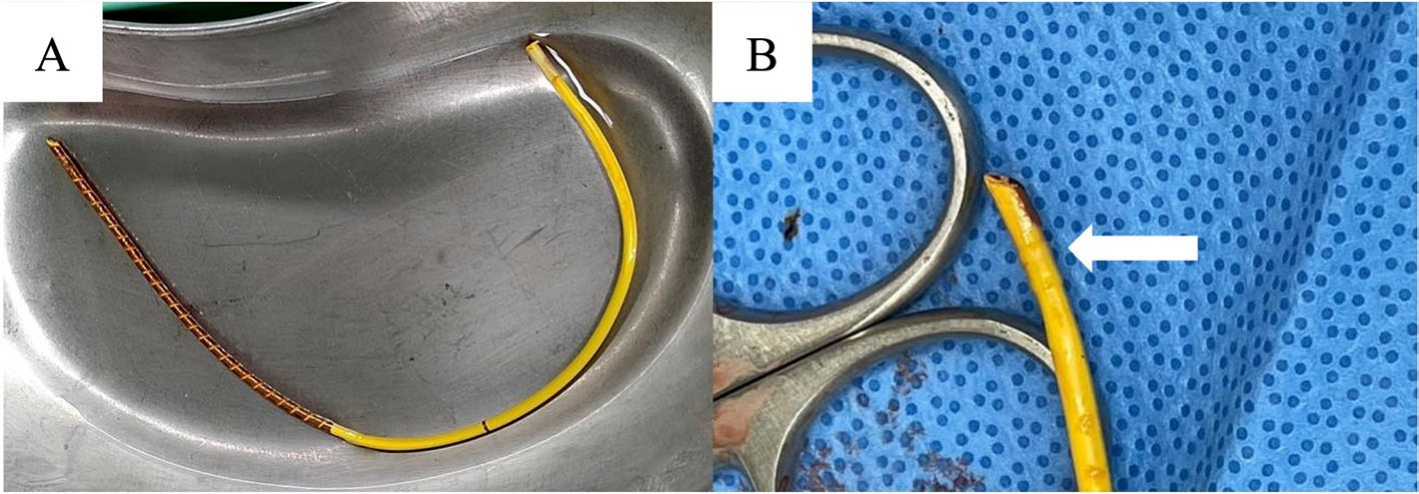
Removed pulmonary artery catheter. The white arrow indicates the site of suture penetration. The catheter was severed intraoperatively to facilitate safe removal

Anesthetic management was uneventful. Patient positioning and surgical approach were identical to the initial surgery. Initially, the suture at the SVC cannulation site was suspected to be the entrapment point and was therefore removed; however, resistance on the catheter persisted. During further exploration, bleeding was difficult to control, and CPB was initiated via femoral arterial perfusion and venous drainage from the right femoral vein and the SVC. Inspection from the right atrium revealed that the left atriotomy suture line in the first surgery had inadvertently penetrated the PAC (Fig. 2A). To facilitate safe removal, the PAC was severed. Once the suture was released, the catheter was removed without resistance (Fig. 3). The balloon cuff appeared intact. The second surgery lasted 5 h and 26 min. Due to intraoperative bleeding, the patient required 6 units of red blood cells, 6 units of fresh frozen plasma, and 40 units of platelets, as determined at the discretion of the attending clinicians. The postoperative course was uneventful, and the patient was discharged from the ICU on postoperative day 4.

## Discussion

In our case, PAC entrapment occurred during minimally invasive mitral valve repair via a right mini-thoracotomy. Intraoperative findings included abnormal pressure waveforms, aspiration of blood from the balloon port, and accumulation of blood in the monitor connector, despite preserved catheter mobility. In the ICU postoperatively, resistance was encountered when approximately 7 cm of the PAC was withdrawn. During reoperation under CPB, the entrapment site was identified at the left atriotomy suture line.

An intraoperative sudden decrease in PAP, with failure to reinflate the balloon and aspiration of blood from the inflation port, should be considered indicative of PAC entrapment [1]. In addition, blood accumulation in the monitor connector has been previously reported in association with the possibility of entrapment by suture or knotting [3–6]. If the PAC is penetrated by a suture, the pressure waveform reflects the pressure at the site of penetration, and blood can be aspirated through the penetrated lumen. If the balloon lumen is penetrated, the balloon cannot be inflated and blood can be aspirated from the balloon inflation port. In contrast, if the catheter is only tightly entrapped by a suture without penetration, or if the needle is inserted yet no leak has developed, different findings would be expected: the pressure may increase, no blood can be aspirated, and the balloon cannot be inflated. Although balloon rupture was a differential diagnosis [7], catheter removal should have been attempted when these findings were detected. In this patient, surgical intervention was required to release the entrapped PAC. The timing of waveform failure might be useful in estimating the suture entrapment point. Furthermore, the abnormal curvature was observed caudal to the tip of the CVC within the SVC, which may have increased the suspicion of left atrial suture entrapment. Additional imaging, such as lateral CXR or computed tomography, might also have been useful in further evaluating this point.

A previous case described pulmonary artery venting catheter entrapment in the right atrium by a stitch placed during left atrial suturing in MICS [8]. The authors noted that the pulmonary artery venting catheter has a larger diameter than a PAC, and that limited access to the surgical field in MICS increased the risk of suture entrapment. We were unable to identify any published cases of PAC entrapment during MICS in the past decade.

An intraoperative image obtained during left atrial closure in the first surgery and a schematic illustration are shown (Fig. 2A, B) [16]. When the PAC was entrapped, its course was presumed to pass through the SVC, right atrium, and right ventricle, with its tip positioned just distal to the pulmonary artery bifurcation. In our case, the mitral valve was exposed through a left atriotomy. Unlike the transseptal approach, this technique does not allow direct visualization of the right heart chambers, which may increase the risk of PAC entrapment. A previous review reported that PAC entrapment was most frequently observed after isolated mitral valve repair, and this mechanism may have contributed to the present case. Furthermore, when the left atriotomy incision is made close to the septum, sutures placed during closure may extend into the right atrium and entrap the PAC. The patient was positioned in the left lateral decubitus position, which may also have influenced the catheter being sutured at the site of left atriotomy closure. These technical aspects of MICS might contribute to the occurrence of this event. Therefore, anesthesiologists and surgeons must remain aware of the possibility of PAC entrapment in MICS, as delayed recognition can result in the need for invasive intervention or lead to serious complications.

A recent review summarized 64 reported cases of PAC entrapment caused by surgical mechanisms during cardiopulmonary bypass. According to the review, 22 cases (34%) involved the superior or inferior vena cava at venous drainage cannula purse-string sites, 14 cases (22%) occurred in the right atrium, including entrapment by atrial cannula sutures, retrograde cardioplegia cannula sutures, or right atriotomy closure lines, and another 14 cases (22%) were associated with the left atrium, such as atrial vent placement or left atriotomy closure, consistent with our case.

According to a recent review, approximately three-quarters of reported cases of PAC entrapment were not recognized until the postoperative period [1]. In our case, entrapment was diagnosed on postoperative day 1 during catheter removal for progression of care, based on resistance and abnormal curvature observed on CXR. Two centimeters of catheter mobility was observed intraoperatively, and resistance was encountered when the PAC was withdrawn 7 cm postoperatively. As some redundancy may exist in the right atrium or right ventricle, catheter mobility alone may not be sufficient to rule out entrapment. Although withdrawal of the PAC by 5–10 cm after weaning from cardiopulmonary bypass has been recommended to detect entrapment [1, 9, 10], the supporting evidence for this practice remains unclear. Complete removal up to the sheath and reinsertion may be the most reliable method; however, it also carries risks and time constraints [11]. Intraoperatively, if entrapment is suspected, TEE may be useful for diagnosis by visualizing the catheter course, assessing its mobility, and identifying any fixation to the cardiac wall [1, 5, 12]. Postoperatively, as in our case, abnormal catheter curvature on CXR may serve as a useful indicator.

Based on our experience and previous reports, the most important consideration during catheter removal is to always keep the possibility of suture entrapment in mind and to avoid forceful withdrawal when resistance is encountered. Even moderate traction has caused tearing of cardiac or vascular structures, resulting in fatal hemorrhage [13]. Continuous reminders and education are essential for preventing and promptly recognizing this complication. Furthermore, PAC entrapment cannot occur if a PAC is not used. The decision regarding invasive hemodynamic monitoring with a PAC should be multidisciplinary, considering its potential impact on intraoperative and postoperative management. For example, in patients without cardiac dysfunction, perioperative PAC use should not be necessary.

In conclusion, we experienced a rare case of PAC entrapment at the left atriotomy suture line during minimally invasive mitral valve repair. In MICS, a left atriotomy approach might be associated with a risk of PAC entrapment. Early recognition of key findings and careful catheter management are essential to prevent serious complications, and the option of not inserting a PAC should always be considered.

## Data Availability

All data are included in this article.

## Abbreviations

PAC: Pulmonary artery catheter
MR: Mitral regurgitation
MICS: Minimally invasive cardiac surgery
CVC: Central venous catheter
TEE: Transesophageal echocardiography
CPB: Cardiopulmonary bypass
SVC: Superior vena cava
CVP: Central venous pressure
PAP: Pulmonary artery pressure
HR: Heart rate
ABP: Arterial blood pressure
ICU: Intensive care unit
CXR: Chest radiography
